# Morgan County Medical Society

**Published:** 1867-06

**Authors:** C. T. Wilbur

**Affiliations:** Secretary


### PROCEEDINGS OF MORGAN COUNTY MEDICAL SOCIETY.

The Morgan County Medical Society held its regular monthly meeting in Freman's Hall, in Jacksonville, on Monday, May 9th.

The meeting was called to order at 11 o'clock A.M., by the president, Dr. R. E. McVey, when the minutes of the last meeting, as printed in the *Jacksonville Journal*, were read and adopted.

The Examining Committee recommended the names of Dr. O. Long, of Jacksonville, Dr. G. A. Wilson, of Virginia, and Dr. J. H. Brown, of Waverly, for membership, when they were unanimously elected.

Dr. J. Rhodes, of Jacksonville, was elected an honorary member of the Society.

Dr. D. A. Logan, of Newmanville, Cass County, J. G. Cox, of Jacksonville, T. N. Stewart, of Exeter, and E. F. Baker, of Alexander, were nominated for membership.

The following notice was passed:

That the corporate members of the Society present be assessed the sum of one dollar and fifty cents for the expense incurred in the Society dinner—and if the amount raised by said assessment exceed the expense thus incurred, the balance shall remain in the hands of the Treasurer, subject to the order of the Finance Committee.

The Society then proceeded by ballot to elect its officers for the ensuing year, with the following result:

*President*—Dr. Henry Jones.

*Vice-President*—Dr. S. Weagley.

*Secretary*—Dr. C. T. Wilbur.

*Treasurer*—Dr. John W. Craig.

In electing the Examining Committee, it was moved by Dr. Prince that the five individuals who received the highest number of votes upon the first ballot should be declared elected.

Drs. Prince, W. S. Edgar, Long, Askew, and Fisher, were elected as the Examining Committee for the ensuing year.

Drs. Prince, Edgar H. Jones, Wilbur, and Craig, were elected delegates to the State Medical Convention.

The following report of the Secretary for the last year was then read:

Gentlemen: Custom makes it incumbent upon me to present a report at the expiration of my term of office. By custom I do not refer to the action of a long line of predecessors, but to what is done by all dignified bodies, like ours, which have Secretaries.

I ought, but shall not be able, to give an exact statistical report of the attendance upon our meetings. Also a volume or two of praise to those who have been punctual and faithful to the Society, and who have labored from the first to make the exercises entertaining and instructive, and a sound lecture to those whose short-comings have been long suffered.

Fourteen meetings have been held during the past year, with a very good average attendance. There are now connected with the Society, thirty-five corporate members, and thirteen honorary members.

Four of the corporate and four of the honorary members reside out of the county.

During the year we have had seven essays read upon the subjects, by the individuals and in the order hereinafter mentioned:

On Cholera, by Dr. H. Jones. Agency of mercury in cholera, diarrhœa, and similar diseases, Dr. D. Prince. On idiocy and its relation to the medical profession, Dr. C. T. Wilbur. On ergot of rye, Dr. W. S. Edgar. On phthisis and its relation with scrofula, Dr. R. E. McVey. On puerperal fever, Dr. D. Prince. On the Calabar bean, Dr. R. Malone.

Our meetings have been enlivened by some very spirited and interesting conversations on the following topics: Ventillation, or the means of changing the viatated air of the sick-chamber. Osteo myelitis. Injections by hydrostatic pressure in acute dysentery. A case of malpractice, introduced by Dr. Prince. Tartar emetic in uterine inertia. Nomenclature and classification of febrile diseases, introduced by Dr. H. Jones. Ergot. Local anesthesia. Introduction of mecurials into the materia medica. Classification and treatment of cutaneous diseases, introduced by W. S. Edgar. Impurities of castor oil, introduced by Dr. Warrinner. Production of insensibility by cold ingeniously applied, introduced by Dr. G. V. Black, who exhibited apparatus. Cerebro spinal meningitis, introduced by Dr. J. P. Johnson.

Some very remarkable and interesting cases occurring in the practice of the various members of the Society have been mentioned and commented upon, of which we cannot in this report make further mention.

A fee bill for the mutual benefit of the members of the Society, to serve as a general guide, was framed and adopted at the meeting held in December, 1866. The reports of the Society, from month to month, have been published in the *Jacksonville Journal*, and several of these reports have been copied into the *Chicago Medical Journal* and EXAMINER—in fact, every report has been published that has been sent to these medical journals by the Secretary. We have in store for the coming year, essays on various topics, promised by Drs. Prince, Henry Jones, H. K. Jones, W. S. Edgar, J. W. Craig, and others, and our meetings, it is hoped, will be still better attended than during the past year. It is also hoped that interesting and rare cases may be reported, and that there may be no lack of topics for conversation for each monthly meeting of the coming year.

Our meetings have been characterized by uniform courtesy of demeanor, one to another, and there has been a complete absence of personalities in our discussions and of any disposition to carry out the common opinion of the public that doctors never agree. On the contrary, the most perfect harmony of action and interchange of good nature have prevailed. In short, our meetings have been such as to cause feelings of pride that we are so generally amiable and fraternizing. There have been no symptoms of intolerance manifested toward the transitory Lighthills, O'Learys, and Finns, and even stationary charlatans have been permitted to bleed their dear public, *ad libitum*,

"With no one nigh to hinder!"

In conclusion, I would respectfully return my thanks for the honor conferred upon me, and the charitable manner in which the imperfect performance of the functions of my office have been received.

The foregoing report is respectfully submitted.

C. T. Wilber, M.D., Secretary.

The Treasurer's report was next read, and both reports were accepted by the society.

Dr. McVey then delivered a spirited and eloquent valedictory address, after which he vacated the Presidential chair, and the newly elected president, Dr. Henry Jones, was conducted to it. Dr. Jones briefly remarked that he was completely unprepared for any remarks, as the result of the election was entirely unexpected by him.

A resolution of thanks for the able manner in which all the duties had been performed by the officers of the last year, was unanimously carried.

The Society adjourned at half-past 1 o'clock to meet at the Dunlap House at 2 o'clock P.M.

At 2 o'clock P.M., the members of the Society assembled at the Dunlap House—where an excellent dinner awaited them. After the invocation of the Divine blessing, by Dr. Johnson, all proceeded to discuss the viands so bountifully spread out before them, and there seemed to be no sign or symptom of impared digestive functions among the whole fraternity.

The members were then called to order by the President, when the Secretary announced the following toast:

"The early practice of Jacksonville," and called upon Dr. Henry Jones to respond.

The doctor then opened the "feast of reason" with the following remarks:

The early practice of medicine in Jacksonville was similar of course to medical life in other new settlements, characterized by hard work, low charges, long credits, and payment in the end, not always, or generally in legal tenders.

Hard work—for in those early days practitioners were nearly all gathered in the larger villages, and the doctor's circuit, if not as broad as the Methodist preacher's, was no narrow one, requiring long rides by night and day, and frequently, long, protracted absences from home; and this work must be performed on horseback, for the doctor's duty led him over routes impracticable to gig or buggy—and any attempt to employ wheels would have ended in sad discomfiture. Just such a life as is now being led in other localities, whose conditions are at present, what ours were some forty years ago.

Yet, in the retrospect of these early times and ways, there is something by no means unpleasant. If the country was young, so was the doctor, for nearly all the practitioners then were young men, and labor was easily bourne, which, I suspect, some of us would shrink a little from encountering now.

And to us from the older States there was something fresh and piquant, and far from unenjoyable in the rough life of the times of which I speak; and I shall not easily forget the warm hospitality, the kindness, and attention to the doctor's comfort and well-being, so continually manifested by my earlier employers in my country rounds. This was characteristic of the time.

We had few classes of doctors then. No homœopathy or hydropathy. No itinerant specialists. No wonderful vagrant curers of sore eyes, or ears, or throats, or other "ills that flesh is heir to."

Besides regular practice, we had nothing but Thompsonianism, or steam practice, and this high pressure affair soon collapsed. If still in existence, it is only probably to be found in operation on some far away frontier.

On the heels of the Thompsonians came the "root doctors,"

also now, I believe, obsolete. These gentlemen called themselves *eclectics*, rather an attractive designation, as it seemed to present the idea that in them was to be found the collected wisdom of all systems carefully separated from what was worthless or erroneous. But the eclecticism of the root doctors consisted in the employment of a few articles from the vegetable kingdom to the exclusion of all other remedial means. The community did not seem to think that they had the root of the matter in them, and they passed away. There *is* a wise eclecticism (not talked about, but employed) which leads the intelligent practitioner to avail himself of whatever of value may be found in any system, and something of value may be found in those most heterodox, and, in general, most absurd.

There is another form of eclecticism developed in later days, in which the practitioner selects his *methodus medendi*, at different times, and in different instances from all systems, no matter how antagonistic to each other, to meet the fantasies or prejudices of the constituency to which he ministers. This, which in another profession, would be like discoursing, in order to meet the requisitions of different creeds, at one time from the Bible, at another from the Koran, and at another from the Hindo Shasters, is confined, I trust, to a very select and limited few. I will not occupy your time in relating incidents illustrating medical life in Morgan County in days long past, all of you probably, even at this day, have an occasional experience of what, in other days formed our daily life; and enough of hard work and rough work still survives the changes of the intervening years.

I have a pleasant remembrance of my early associates in practice in this county. They were intelligent, educated men, and entered with good will and energy into the life-work which they had chosen. But one of that number remains in the field, and he is here on this occasion. Some are dead, others have removed to other scenes. I should be glad to say a word in regard to them, and others who, like them, have occupied the position of pioneer doctors in the new settlements of the country. I hesitate, lest I should be thought to include myself in the number of those to whom I wish to pay a tribute as sincere as well deserved. If you will exonorate me from the imputation of such inexcusable egotism and vanity, for I detach myself from the number of those of whom I wish to speak, I will go on. What I wish to say is this—that I cannot look on the lives of these early practitioners without feelings of emphatic admiration, and with a strong conviction that their humble, obscure, laborious, self-sacrificing, and useful lives deserve a higher appreciation than it will ever be their lot to receive. With great labor, with no prospect of fame, with little expectation of more than a bare support; with much self-denial, and often with much difficulty from year to year "in making the two ends meet," their best services were cheerfully rendered; and to many a remote and lonely cabin, the homely-garbed doctor, plodding patiently on from dwelling to dwelling, was, if not in aspect, in reality, an angel of mercy and a ministering spirit. Whatever his reward, it certainly was not of this world, but his memory will be green in the hearts of some who were subjects of his care. All laud and honor, say I, to these humble workers in this noble calling, and let them not be forgotten when they are seen no more.

In response to the toast, "Be short," Dr. David Prince remarked: "The whys and wherefores of the above sentiment I am really at a loss to understand. Physically I am neither short nor long. I am not aware that I am even accused of being "short" in professional or pecuniary relations to society. I must, therefore, pause, as I can conceive of no adequate reason why I should be called upon to reply to this sentiment.

[The effect of the doctor's sentiment was a very happy one, as no one dared to be very prosy.]

Dr. R. E. McVey was called upon to narrate the "pleasures of a country practice," which he did in a very brief manner.

In response to the toast "The Morgan County Medical Society," Dr. W. S. Edgar made the following remarks:

If nature holds no anniversaries, she marks time by the revolution of the seasons, and we are reminded by the recurrence of spring-time of the organization of our Society one year ago.

In adopting a name, the friends were not unmindful of the fact that Morgan County had been one of the most prominent and highly favored counties in the State from its first settlement. Indeed, while yet the smoke curled gracefully over the wigwams of Keokuk, Wapello, and Blackhawk, the pioneers of education appeared with those of civilization to appropriate the choice lands and localities to that high interest; and to their efforts at that early day, are we chiefly indebted for the leading part her sons have ever bourne in the eventful history that has since transpired, as well as for the truly enviable reputation our county and city has enjoyed for its appreciation of education and true progress. In agriculture she has boasted the chief of farmers, whose fame as such was world-wide, leaving an enduring monument of individual enterprise and achievement.

As there is much in a name, we presume the truly enviable history of the county might contribute somewhat to the success of our enterprise, which did not begin its existence without misgivings on the part of its friends, lest through the indifference of some and inconvenience of other members of the profession, it might fall "still born," or, at least not survive its dentition. But, as more hopeful councils prevailed, we here to-day verify, not simply the practicability, but satisfactory success of the experiment.

The rapid advancement in all departments of medical sciences the past half century, we have reason to believe is mainly due to the numerous organizations of this character in our country and the civilized world.

While other medical schools initiate the novice into our profession, the school for thesis and debate cultivates and qualifies him for its arduous duties.

The observations and theories which find their way into Medical literature through this crucible, are generally worthy the attention of the student of his profession. We are aware that there are medical gentlemen, of pretention, too, who, by a habit of non-intercourse with their neighbors, in consultation or discussion, foster the somewhat popular idea, that to be born the seventh son, or "in the light of the moon," is to have medical knowledge by intuition which is preferable to the teaching of books or societies. But as few of us can boast these great natural advantages, medical societies are needed to impart knowledge in the regular and legitimate way, to the uninspired at least, while the study and practice of the medical profession, affords the means of the highest culture and development, both of the moral and intellectual man, when pursued with that end. If pursued for selfish, mercenary ends, and popularity in the profession, with an extensive practice, is attained as the fruit of a system of general demagoging, good Lord, and good devil, good Christian, good infidel, good saint, good sinner, good homœopath, good allopath, all things to all men to gain money, it certainly furnishes the occasion for cultivating the most undesirable character we can conceive.

On the contrary, if the extended acquaintance and practice comes as the fruits of the successful treatment of disease, from laborious study of the profession and valuable contributions to medical science, it is truly an honor and satisfaction to enjoy such public confidence.

As the practice of our profession necessarily leads to a competition of a peculiar and delicate character, it is desirable that our social and friendly relations be such that a generous hand may ever be ready to cast the mantle of charity over any seeming dishonor or error of a professional brother; and as a means to this end we unite around the social board on this anniversary occasion.

In response to the toast, "Southern Practice," Dr. O. M. Long remarked that he would postpone any extended remarks until some future occasion.

Dr. Rhoades responded to the sentiment, "Vicarious Finger Sight," with a few brief remarks.

Dr. Askew, in response to the sentiment, "The Profits of Practice," remarked that, "if any practitioner of medicine hoped to make a fortune in the legitimate practice of his profession, he was afraid he would be doomed to disappointment."

Dr. Fisher, in response to the sentiment, "Experience *versus*

Theory," remarked that "He could not understand why, when there were those present who had been much longer in the field, that he should be called upon to talk of experience. Though he deemed theory very important, yet experience was quite as essential to a successful practice."

Dr. M. M. L. Reed gave a very thrilling narrative of "the difficulties of medical practice in the early settlement of Illinois."

Dr. Johnson, of Lynnville, in response to the sentiment, "The farm the fruit of a successful practice," remarked: "I have the farm, and I will tell you how I got it, and how you may do likewise. Forty years ago, I pledged myself to total abstinence from all intoxicating drinks. I also made it a rule never to be away from my office unless called away by professional business. By strict adherence to these two rules he deemed that a farm, if desired, would follow as the fruit of a successful practice."

Dr. J. T. Cassell, in response to the toast, "Early Practice in Kentucky," remarked that "he believed that in the legitimate practice of medicine a practitioner could acquire a fortune. In a few years' practice in Kentucky, by diligence and hard service, he had secured enough to supply his wants for the balance of his days."

Dr. J. W. Craig, of Arcadia, in response to a sentiment read a brief and very humorous poem, which was received with great applause.

Dr. G. R. Bibb made a few remarks upon "The Tribulations of a Young Practitioner."

Dr. Lucus responded to the sentiment, "Medical Science at home and abroad," and called out Dr. Prince.

Dr. Prince made a few remarks and proposed "The Records of the Morgan County Medical Society," calling Dr. Wilber to respond.

Dr. Wilber remarked that having taken up so much time of the Society in his report at the morning session, he would simply in the name of the Society offer a conundrum:

"Why are the frequent non-attending members of the Society like gophers?" Because they make their marks here and there in little mounds of fresh earth, but that is all the Society sees of them.

Dr. DeLeuw gave in response to "Change of Location," the sentiment, "a '*Pereat!*' to all who pretend to be physicians, but who are *only* pretenders."

Dr. Knight spoke in response to the sentiment, "Practice in Town and Country," and Dr. Weagley in response to "Medicine and Agriculture.,,

Dr. Buckner suggested the propriety of adopting more method in the appointment of individuals to take part in the regular meetings of the Society.

His remarks resulted in the passage of a motion instructing the Secretary to call upon the members in alphabetical order to prepare essays for the regular meetings of the Society.

Drs. Askew and Buckner were announced by the president as the essayists for the next regular meeting.

At 5 o'clock P.M., the Society adjourned to meet on the second Thursday in June.

C. T. WILBUR, M.D., Secretary

